# Tobacco consumption, sales, and output as monitoring indicators in the era of the tobacco endgame: a Korean example

**DOI:** 10.4178/epih.e2023030

**Published:** 2023-02-27

**Authors:** Hana Kim, Hee-kyoung Nam, Heewon Kang

**Affiliations:** 1 Department of Public Health Science, Graduate School of Public Health, Seoul National University, Seoul, Korea; 2 Institute of Health and Environment, Seoul National University, Seoul, Korea

**Keywords:** Tobacco use, Tobacco output, Tobacco sales, Tobacco endgame, Monitoring, Drug trafficking

## Abstract

**OBJECTIVES:**

The consumption, sales, and output of tobacco products each suggest different areas of intervention for tobacco control. In the era of the tobacco endgame, as increasingly stronger supply-side measures are implemented, multifaceted indicators that assess both supply and/or demand are required. We aimed to estimate the consumption of cigarette and heated tobacco products (HTPs) and sought agreement between the various indicators.

**METHODS:**

The annual cigarette and HTP consumption in 2014-2020 was calculated using the frequency and intensity of cigarette use from representative surveys of adults and adolescents by sex and age. Sales and output data were acquired from governmental sources. Spearman correlation coefficients and Bland–Altman plots were used to compare the indicators.

**RESULTS:**

Tobacco output, cigarette sales, and cigarette consumption were greatest in 2014. The HTP consumption calculated for 2020 was 292.28 million packs. Cigarette consumption and sales correlated significantly, as did tobacco output and tobacco sales. A Bland–Altman plot comparing the difference between cigarette consumption and sales showed that this difference was largest in 2014, immediately before cigarette prices increased. With the exception of a single year, all cigarette consumption values were within the limits of agreement for cigarette sales and tobacco output.

**CONCLUSIONS:**

Our analyses showed agreement between demand-side (tobacco consumption) and supply-side (sales and output) indicators. We recommend using all indicators to monitor the impacts of tobacco control on both demand and supply sides. The systematic use of various indicators is critical to achieve the end of the tobacco epidemic.

## GRAPHICAL ABSTRACT

[Fig f5-epih-45-e2023030]

## INTRODUCTION

A growing number of countries have set targets and planned innovative tobacco endgame actions to finally end the tobacco epidemic. The current monitoring system focuses on traditional demand indicators, such as smoking prevalence and tobacco consumption. However, the tobacco endgame focuses on supply-oriented strategies, such as the introduction of retailer licensing and bans on outlets and product displays [1]. In this era of the tobacco endgame, the monitoring and supplementation of various smoking indicators are important to identify and regulate the manufacture and distribution of tobacco products.

The tobacco output is the amount of tobacco moved from factories, excluding exports and in-factory disposal; tobacco manufacturers predict tobacco production and retailers provide tobacco to consumers from manufacturers’ output [2]. In addition, the scale and budget of national health promotion projects are estimated based on tobacco output [4]. The Korean Ministry of Health and Welfare has stated that the output reported by tobacco manufacturers and import companies serves as an official indicator of tobacco consumption [5].

Tobacco consumption generally refers to the amount of smoking, calculated using self-reported survey data [6,7]. In Great Britain, tobacco consumption was defined as the smoking amount obtained from self-reported surveys, despite well-known limitations such as low response rates, recall bias, and limitations of the survey sampling design [3]. Smoking prevalence is very useful for monitoring a population’s tobacco use. However, information could be overlooked when measuring smoking prevalence if tobacco consumption is not also used. For example, one person smoking more than 20 cigarettes per day and another smoking 1 cigarette per week would be judged equally as current smokers, with no consideration of the magnitude of their smoking habits, when measuring smoking prevalence [8]. Thus, the investigation of tobacco consumption with smoking prevalence in a population is important [8].

Retail sales data are also used in the United States, Ireland, and the United Kingdom to track the populations’ tobacco use [3]. As sales data are objective, they are generally considered to be a stronger alternative than other indicators for the estimation of smoking [3]. Sales data have been used in many countries, and they provide evidence supporting the enhancement of national tobacco control policies for the monitoring and evaluation of this status [3]. The Food and Agriculture Organization of the United Nations reported on tobacco production in 168 countries from 1961 to 2009, and the U.S. Department of Agriculture released a tobacco yearbook documenting the amount of tobacco manufactured in 165 countries from 1960 to 2005 [9]. Euromonitor also released international cigarette sales data from 1998 to 2012, including retail and illegal trade data [9].

We compared tobacco-related indicators in Korea to illustrate the importance of the use of various indicators worldwide. Korea achieved the highest level for collecting and monitoring data on tobacco use [10]. In addition, many national tobacco use surveys of adults and adolescents in Korea have been conducted; they include the Korea National Health and Nutrition Examination Survey (KNHANES), the Social Survey, the Korea Community Health Survey (KCHS), and the Korea Youth Risk Behavior Survey (KYRBS) [11]. The output and sales of cigarettes and heated tobacco products (HTPs) are regularly monitored under the government’s lead [12].

The monitoring of populations’ tobacco use has become complex with the advent of new tobacco products. With the industry’s gradual diversification, new survey items have been introduced to encompass novel tobacco products. Questionnaire items about nicotine products were introduced in the 2013 KNHANES and 2011 KYRBS, and those about HTPs were introduced in the 2018 KYRBS (ever used) and 2019 KNHANES (ever used/current use) [11]. The quantity of HTPs consumed was also investigated in the 2020 KYRBS and the 2019 KNHANES. After the introduction of HTPs in Korea in June 2017, HTP sales have been reported by the Ministry of Economy and Finance (MOEF) [12].

Thus, the accurate monitoring of smoking status and the identification and comparison of indicators reflecting the tobacco distribution process are important for the implementation of an endgame strategy. We aimed to estimate the consumption of cigarettes and HTPs and assessed whether demand-side indicators (consumption) and supply-side indicators (output, sales) were comparable.

## MATERIALS AND METHODS

### Self-reported use of tobacco products (cigarettes and heated tobacco products)

Data from the KNHANES for adults and the KYRBS for adolescents were used to identify tobacco consumption patterns by the Korean population. The KNHANES and KYRBS are population-based nationwide cross-sectional surveys conducted annually by the Korea Disease Control and Prevention Agency to monitor the health behaviors of Koreans [13,14]. The average participation rates were 75.90% (range, 74.00 to 78.30) for KNANES and 95.99% (range, 94.90 to 97.20) for KYRBS. Tobacco consumption was estimated using data from 2014-2020 for comparison with the sales and output data, and HTP consumption was only estimated for 2020, as data on the frequency and quantity of HTP use by both adults and adolescents were only available for 2020.

### Tobacco product output and sales

The MOEF has released monthly tobacco output and quarterly sales data since 2014 in a report called “Trends in the Cigarette Market” [12]. In this report, sales of various tobacco/nicotine products are released, but only cigarette and HTP data were used in this study. These data were compared with the estimated tobacco consumption of Koreans since 2014. The collection of monthly data on HTP sales began in June 2017, with the market introduction of the products [12]. As it was difficult to place the HTP outputs into 1-year units in 2018 and 2020, the released tobacco (cigarettes and HTP) outputs were used in this study.

### Statistical analysis

Annual cigarette and HTP consumption were calculated with the classification of smokers as daily and occasional smokers by sex and age. Consumption was calculated separately for adults and adolescents and summed. Cigarette consumption by daily smokers was calculated as 12 × 30 × smoking quantity per day. Cigarette consumption by occasional smokers was calculated as 12× number of smoking days (during the last month)× smoking quantity per day. Cigarette packs were defined as containing 20 cigarettes, and the consumption unit was converted to 1 million packs for sales and output comparisons [6].

As the number of cigarettes smoked was a continuous variable in the KNHANES, the calculated amounts of cigarettes and HTPs consumed were summed using weighting and the PROC SURVEYMEANS procedure in the SAS version 9.4 (SAS Institute Inc., Cary, NC, USA). As the numbers of smoking days and cigarettes and HTPs consumed were categorical variables in the KYRBS, we recategorized the data using the mean of the response categories. Specifically, the responses to the question about smoking quantity (“How many cigarettes/HTPs did you smoke/use per day on average in the last 30 days?”), which originally were < 1 cigarette/day, 1 cigarette/day, 2-5 cigarette/day, 6-9 cigarette/day, 10-19 cigarette/day, and ≥ 20 cigarette/day, were reclassified as 0.0 cigarette/day, 1.0 cigarette/day, 3.5 cigarette/day, 7.5 cigarette/day, 14.5 cigarette/day, and 20.0 cigarette/day, respectively. Furthermore, the responses to the question about the number of smoking days (“How many days have you smoked/consumed HTPs in the last 30 days?”), which originally were less than 1 day/mo, 1-2 day/mo, 3-5 day/mo, 6-9 day/mo, 10-19 day/mo, 20-29 day/mo, and every day, were reclassified as 0.0 day/mo, 1.5 day/mo, 4.0 day/mo, 7.5 day/mo, 14.5 day/mo, 24.5 day/mo, and 30.0 day/mo, respectively.

The analyses employed SAS, R, and Excel software; all figures were drawn with the aid of R version 4.1.1 (R Foundation for Statistical Computing, Vienna, Austria). Non-parametric Spearman rank analyses were used to seek correlations between indicators. Bland–Altman plots were drawn to explore the extent of agreement between pairs of indicators [15,16]. The results from the Bland–Altman analyses are rather robust regardless of data normality [17]. We performed 2 sensitivity analyses; the methods and results are detailed in Supplementary Material 1.

### Ethics Statement

This study was exempted from review by the Institutional Review Board of Seoul National University (IRB No. E2212/002-003).

## RESULTS

**Figure 1.**
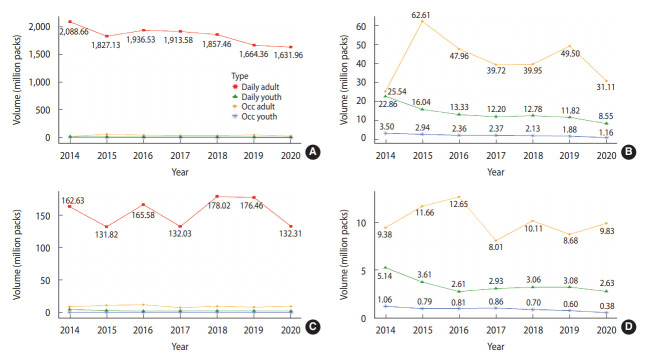
Estimated cigarette consumption by daily and occasional (Occ) smokers by sex. (A) Total male cigarette consumption by type. (B) Cigarette consumption among Occ male smokers and boys. (C) Total female cigarette consumption by type. (D) Cigarette consumption among Occ female smokers and girls.

Annual cigarette consumption by sex from 2014 to 2020 is shown in Figure 1. Regardless of smoking frequency or age, cigarette consumption was higher in males than in females. Both males and females who smoked regularly made significant contributions to consumption. For both sexes, the trends in cigarette consumption over 6 years were similar, but the cigarette consumption of daily male smokers decreased from 2017 to 2018, whereas that of daily female smokers increased from 2017 to 2018. In particular, occasional smoker patterns fluctuated more frequently in females than in males. Cigarette consumption by daily and occasional adolescent smokers was highest in 2014 and lowest in 2020.

**Figure 2.**
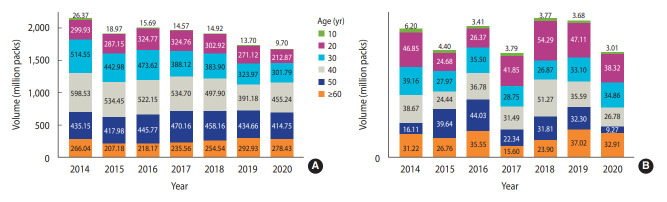
Estimated tobacco consumption by sex (A: males, B: females) and age.

The estimated cigarette consumption by sex and age is shown in Figure 2. Males in their 40s evidenced the highest cigarette consumption in all years except 2019. Females in their 50s smoked the most cigarettes in 2015 and 2016, but in the other years, females in their 20s had the highest consumption.

**Figure 3.**
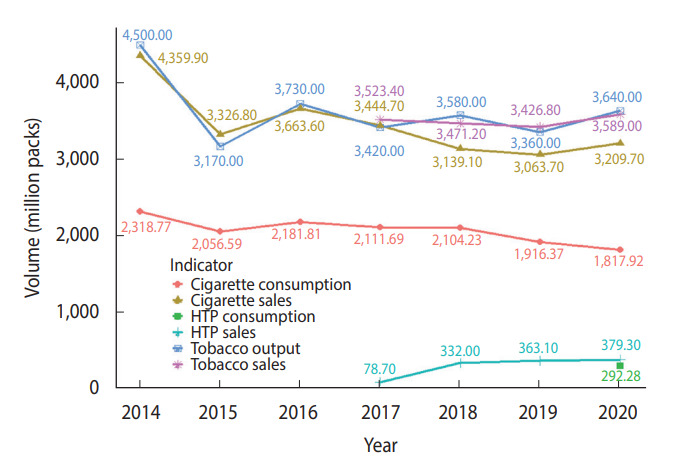
Comparison of tobacco (cigarette and HTP) consumption, sales, and output during 2014-2020. HTP, heated tobacco products.

The estimated annual consumption, sales, and outputs of tobacco from 2014 to 2020 are shown in Figure 3. The difference between cigarette consumption and sales was largest in 2014 (n= 2,041.13 million packs). The trends in annual cigarette consumption and sales were similar from 2014 to 2019. However, whereas cigarette consumption declined from 2019 to 2020, cigarette sales and tobacco sales and output increased. The trend in HTP sales increased from 2017 to 2020. In 2020, about 292.28 million packs of HTPs were consumed and roughly 379.30 million packs of HTPs were sold. The difference between HTP sales and consumption in 2020 was estimated to be 87.02 million packs.

Correlations between cigarette consumption, cigarette sales, tobacco sales, and tobacco output from 2014 to 2020 are shown in Table 1. The Spearman coefficient of correlation between cigarette consumption and sales was 0.82 (p=0.034), and that between tobacco sales and output was 0.96 (p= 0.003).

**Figure 4.**
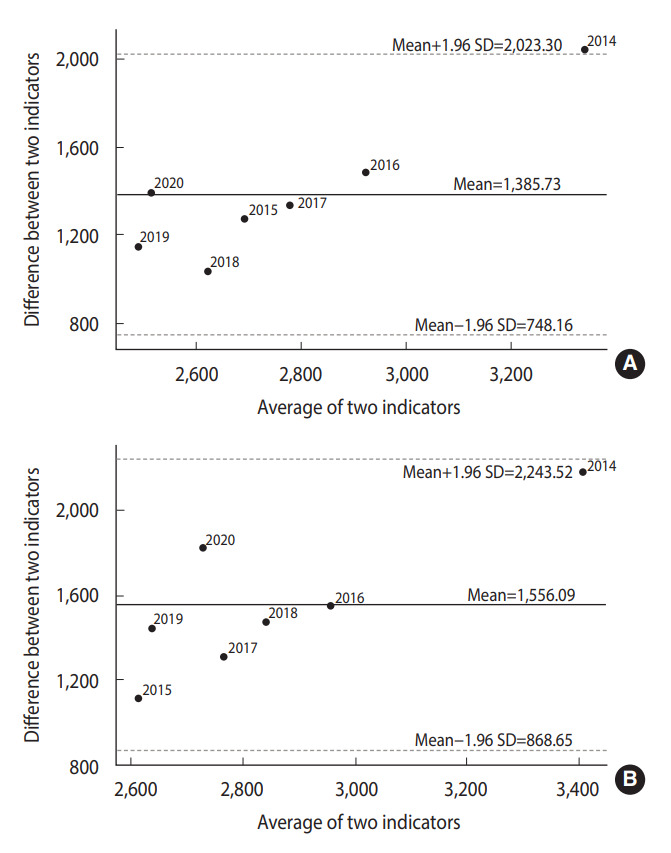
Bland–Altman plots of differences between cigarette consumption and other indicators. (A) Cigarette consumption*Cigarette sales. (B) Cigarette consumption*Tobacco outputs. SD, standard deviation.

The extent of agreement between pairs of indicators is shown as Bland–Altman plots in Figure 4. The difference between the cigarette consumption and sales values was largest in 2014, when the average of the indicators was 3,339.33, outside the limit of agreement (Figure 4A). When the average was less than 3,000, the values were distributed close to the mean of the differences between the two indicators (mean, 1,385.73). The 95% confidence interval (CI, representing the limits of agreement), calculated using the formula of mean± 1.96 standard deviations, was 748.16-2,023.30. The difference between cigarette consumption and tobacco output was distributed near the average for all years except 2014 (Figure 4B). When the average was 3,409.38, the difference between the two indicators was far from the mean (mean, 1,556.09). All values were within the limits of agreement. The 95% CI was 868.65 to 2,243.52.

## DISCUSSION

We estimated annual cigarette consumption from 2014 to 2020 and HTP consumption in 2020 and compared the results with sales and tobacco output data. More than 86% of the total estimated cigarette consumption each year was attributable to daily cigarette consumption by male daily smokers. The difference between annual cigarette consumption and sales was smallest in 2018 and largest in 2014. The trend in cigarette consumption remained similar to that in cigarette sales from 2014 to 2019 but showed a different pattern from 2019 to 2020. Specifically, cigarette sales increased while consumption decreased from 2019 to 2020. The difference and average values between annual cigarette consumption and tobacco output were distributed within the 95% CI, confirming agreement. However, the value for the difference between cigarette consumption and sales in 2014 fell outside of the CI. Hoarding of cigarettes by smokers was reported before the largest price increase occurred in 2015 [18].

The analyses of consumption volumes by sex and age highlighted the following points. Decreasing trends in tobacco consumption were evident for males, but the trends fluctuated for females, possibly indicating that consumption estimates were biased by social desirability. The decreases in tobacco consumption were most marked from 2014 to 2015 for males and from 2019 to 2020 for females. Tobacco consumption in males fell the most in 2015 compared to the previous year, when the price increased, consistent with previous findings [19,20]. Recent increasing consumption trends are evident in females in their 20s and 30s.

Gallus et al. [21] reported a gap between self-reported results and sales, consistent with our results and this gap has been explained by smuggling, social unacceptability, immigrant use, hand-rolling of cigarettes, and methodological sampling and survey problems. Similarly, the gaps between trends in the indicators examined in this study might be due to temporal differences in tobacco distribution process, the exclusion of subjects from surveys, differences in evaluation methods, and changes in survey questions.

The temporal gap between tobacco distribution and investigation may have resulted in differences between the smoking indicators. The MOEF announces quarterly tobacco output and cigarette sales, and the KNHANES and KYRBS are conducted only annually. Thus, the timing of consumer purchase, distribution and sales reporting, and investigation of cigarette consumption may have differed. Specifically, the difference between cigarette consumption and sales may have been caused by cigarette hoarding/storage at the time of the survey, or by the hand-rolling of cigarettes and illicit transactions. The difference between tobacco output and sales may have been caused by the temporal difference in distributors’ delivery of cigarettes to retailers [4].

The exclusion of some subjects from the KNHANES and KYRBS may also be relevant. Soldiers and foreign residents are excluded from the KNHANES [22]. Moreover, the KYRBS did not examine the smoking prevalence among or tobacco consumption of outof-school youth [23]. For example, smoking was more prevalent among soldiers than among males in the general population aged 19-29 years from 2012 to 2017 [24]. The smoking prevalence among soldiers, who are estimated to contribute significantly to the three missing populations, was 37.9% in 2019 [25], when the total number of soldiers was 579,000 [26]. Assuming that such smokers (n= 219,441) consumed 10 cigarettes per day, they would have contributed about 2,194,410 cigarettes to the national daily tobacco consumption. The identification of such blind spots in national surveys is essential for accurate monitoring of smoking status and implementation of an endgame strategy.

Survey data are particularly affected by methodological issues, under-reporting, and bias. The survey data were not reflected accurately in the annual cigarette consumption data, leading to gaps in the indicators. In particular, smoking among females and adolescents could be underreported for socio-cultural reasons [27,28]. Self-reported smoking status is also subject to under-reporting or over-reporting due to recall bias [29]. In particular, this bias may affect elderly individuals’ responses regarding specific periods in health behavior and medical use surveys [30]. Non-respondent bias may also affect the estimation of tobacco consumption from survey data. For example, according to Liber & Warner [7], reliable data collection in the United States is difficult because the proportion of non-responders has increased steadily over the past half-century. Thus, annual self-reported surveys must be conducted with consideration of the characteristics of the target population and complemented with objective smoking indicators, such as sales and output.

Another limitation of survey data is that tobacco consumption estimates vary depending on the survey question response structure and the institution conducting the survey. In this study, as the response to the KYRBS question about the number of cigarettes smoked per day was categorical, mean values were imputed, which may have led to the over-estimation or under-estimation of the annual tobacco consumption of adolescents. To investigate the smoking status of adolescents more accurately, survey responses should be direct, rather than categorical. In Korea, health surveys conducted by various institutions have different items, evaluation methods, and survey subjects [6]. Thus, the comparison of cigarette consumption calculated from other survey data, such as those from the Social Survey or KCHS, is needed in future studies.

Finally, changes in survey questions may have affected tobacco consumption estimates. The calculated cigarette consumption was less in 2019 and 2020 than before 2018, which may reflect the changes to the KNHANES and KYRBS questions about smoking to encompass new tobacco products [31]. The use of recently emerged novel tobacco products is difficult to reflect in surveys. Such failure to accurately measure tobacco consumption can be compensated for by the use of indicators such as sales and output.

The reasons for the difference between HTP consumption and sales in 2020 may be similar to those for the difference between cigarette indicators. The former was smaller than the latter, which may falsely report tobacco outputs [32]. Thus, rigorous monitoring must continue. Recall bias likely contributed less to the difference between the HTP indicators than to that between cigarette indicators because most HTP users are young. The recent emergence of new tobacco products could lead to errors in self-reported surveys due to the lack of detailed terminology. Hence, descriptions and information about all tobacco products assessed must be added to survey questions [33].

Significant correlations and considerable agreement between the smoking indicators were confirmed in this study. This finding indicates that not only cigarette consumption but also sales and output are indicators that reflect a population’s smoking status. The Unite States state alcohol consumption and sales from 1993 to 2006 estimated from survey data accounted for 22-32% of annual sales, and these variables were strongly correlated [34]. Sales data do not imply actual consumption because people can store beers without drinking them, and smuggling and consumption by tourists and soldiers were excluded from the calculations [34]. These explanations may be equally applicable to tobacco consumption.

The comparison of cigarette indicators such as consumption and sales may be useful for the determination of the effectiveness of tobacco control policies [35] and identification of hoarding [36]. Tobacco consumption in Indonesia exceeds local production, indicating that imports have increased due to low tobacco import tariffs and a lack of enforcement of the tobacco control policy [37]. If only the smoking prevalence had been surveyed in Indonesia, the need to strengthen tobacco control policies and the cause for the increase in the smoking population would not have been identified. Tobacco outputs were less than tobacco sales in Korea in 2017, suggesting that tobacco manufacturers and importers sold more cigarettes to consumers than they distributed on the market. The difference between the 2 indicators suggests that retailers hoarded cigarettes following the introduction of the cigarette pack warning picture in Korea in December 2016 [38]. Sales were higher than outputs in 2019, which might be interpreted as cigarette hoarding by retailers due to the government’s announcement of the strengthening of non-price policies.

In 2019, the Korean government announced a plan to establish a national tobacco supply management system to prevent illegal tobacco product transactions [39]. The Eliminate Illicit Trade in Tobacco Products protocol was adopted unanimously at the 5th General Assembly of the World Health Organization Framework Convention on Tobacco Control in 2012 [40]. The core contents of this protocol included the establishment of national tobacco distribution tracking systems and regulation of the tobacco product supply chain [40]. Stamp programs and digital codes have been used to track illegally traded tobacco products in Uganda, the United Kingdom, California, and Brazil [40]. The application of such measures or monitoring of various indicators, such as output and sales together, could help to prevent illicit transactions.

The monitoring of various indicators is essential when establishing national health promotion plans, such as Healthy People in the United States and health plans in Korea, and when introducing an endgame strategy. In particular, illegal trade could increase following the introduction of such a strategy. Therefore, exports, imports, sales, and output should be used as indicators of illegal trade.

A limitation of this study was that gaps existed between sales, output, and estimated consumption from survey data. Some of the gaps were narrowed by the two sensitivity analyses, which estimated tobacco consumption by the missing population and the time lag between tobacco supply and demand, but gaps remain. Further research on the life cycles of tobacco/nicotine products, including manufacture, importation, distribution, and consumption, is required. However, the trends in the indicators were similar, and the validity of the results was confirmed through the correlation and Bland–Altman analyses. In addition, limitations were found in the current reporting system focusing on smoking prevalence. This study provides evidence supporting the establishment of a monitoring system and tobacco control policies centered on the distribution system, and it could provide direction for the accurate portrayal of the population’s smoking status.

We demonstrated that the indicators for the demand and supply sides were within the limits of agreement. The indicators that we investigated are all imperfect if used alone, but indicate the impacts of different interventions. For example, the effectiveness of smoking cessation assistance or smoking prevention education is reflected in consumption. Restriction of tobacco retailer licensing affects sales. It is important to select and evaluate indicators that reveal whether policies are working. The investigation of the tobacco use of foreigners, soldiers, and out-of-school youth in representative surveys is necessary. We must develop and complement survey items in synchrony with the introduction of new tobacco products. Above all, demand and supply indicators should be used together from an integrated system perspective for monitoring. Various tobacco/nicotine use indicators other than prevalence, such as consumption, output, and sales, should be used to end the tobacco epidemic and regulate illegal tobacco product transactions.

## Figures and Tables

**Figure f5-epih-45-e2023030:**
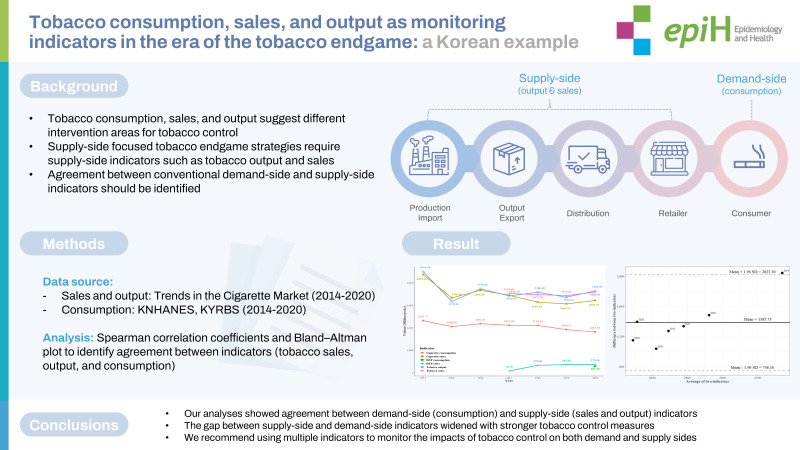


**Table 1.** Correlation between trends in indicators

Variables	Tobacco output	Tobacco sales	Cigarette sales	Cigarette consumption
Tobacco output	1.00			
Tobacco sales	0.96	1.00		
p-value	0.003			
Cigarette sales	0.61	0.71	1.00	
p-value	0.167	0.088		
Cigarette consumption	0.57	0.61	0.82	1.00
p-value	0.200	0.167	0.034	

## SUPPLEMENTARY MATERIALS

Supplement Material 1. Sensitivity analyses
