# Supplementary material for: Tobacco consumption, sales, and output as monitoring indicators in the era of the tobacco endgame: a Korean example
Source: Epidemiol Health. 2023 Feb 27;45:e2023030. doi: 10.4178/epih.e2023030 (PMC10396800; doi:10.4178/epih.e2023030)
Supplement: Supplement Material 1. — Sensitivity analyses [file epih-45-e2023030-Supplementary.pdf]

Supplement 1. Sensitivity analyses

(A) Application of missing population

Missing population (Year surveyed)	Population	Assumed smoking prevalence	Cigarette consumption (/cigarette)	Cigarette consumption (/million packs)
Out of school (2014)	51,906	59.50%	30884.07	0.00154
Foreigners (2020)	2,146,748	10%	214674.80	0.01073
Soldiers (2019)	579,000	37.90%	219441	0.01097
Cigarette consumption of missing population			464999.87	0.02325

Year	Surveyed cigarette consumption (/million packs) (1)	Estimated cigarette consumption of missing population (/million packs) (2)	(1) + (2)
2014	2318.77	0.02325	2318.79
2015	2056.59	0.02325	2056.61
2016	2181.81	0.02325	2181.83
2017	2111.69	0.02325	2111.71
2018	2104.23	0.02325	2104.25
2019	1916.37	0.02325	1916.39
2020	1817.92	0.02325	1817.94

We estimated the cigarette consumption of the missing population in the national representative health behavior surveys and presented it in Supplement 1(A). We combined the missing population's tobacco consumption with the surveyed population's tobacco consumption.

Korea had 2,146,748 foreign residents on November 1, 2020. Assuming that 10% of these individuals consumed 10 cigarettes per day (total = 214,675), they contributed about 2,146,750 cigarettes to the population's tobacco consumption. Although out-of-school youth have a high risk of exposure to smoking and relatively weak control over smoking, their smoking status was not investigated in the KYRBS[1]. In 2014, 51,906 students were not enrolled in school[2] and 59.5% of them smoked every day[3]. Assuming that smokers (N = 30,884) smoked 10 cigarettes per day, their contribution to the population's tobacco use was approximately 308,840 cigarettes.

(B) With and without time lag (a year)

	With time lag					Without time lag				
**Year**	**Cigarette consumption**	**Tobacco output**	**Cigarette sales**	**Tobacco output – cigarette consumption**	**Cigarette sales – cigarette consumption**	**Cigarette consumption**	**Tobacco output**	**Cigarette sales**	**Tobacco output – cigarette consumption**	**Cigarette sales – cigarette consumption**
**2014**		4500	4359.9			2318.77	4500	4359.9	2181.23	2041.13
**2015**	2318.77	3170	3326.8	851.23	1008.03	2056.59	3170	3326.8	1113.41	1270.21
**2016**	2056.59	3730	3663.6	1673.41	1607.01	2181.81	3730	3663.6	1548.19	1481.79
**2017**	2181.81	3420	3444.7	1238.19	1262.89	2111.69	3420	3444.7	1308.31	1333.01
**2018**	2111.69	3580	3139.1	1468.31	1027.41	2104.23	3580	3139.1	1475.77	1034.87
**2019**	2104.23	3360	3063.7	1255.77	959.47	1916.37	3360	3063.7	1443.63	1147.33
**2020**	1916.37	3640	3209.7	1723.63	1293.33	1817.92	3640	3209.7	1822.08	1391.78
	1817.92									

We used a time lag of 1 year to find the difference between supply and consumption and presented it in Supplement 1(B). Except for 2016, the difference between supply and consumption in all years with a time lag decreased, compared to without a time lag.

Reference

1. Bae JE, Kim C-W, Lee SE, Im H-B, Kim IY, Lee T-Y, et al. Factors related to the smoking relapse of out-of-school adolescents. Korean Journal of Health Education and Promotion. 2021;38(3):13-21.

2. Ministry of Education. The Survey on the Status of Out of school Students in 2014 (as of '15.4.1)("Korean, author's translation"). 2015 Available from: https://www.korea.kr/briefing/pressReleaseView.do?newsId=156073677.

3. Geun-Soo Park, Min Kim. A Comparative Study of Health Status between Students and Out-of-School Youth. Korea institute of youth facility and environment. 2016;14(2):17-26.
